# Osmolytes: A Possible Therapeutic Molecule for Ameliorating the Neurodegeneration Caused by Protein Misfolding and Aggregation

**DOI:** 10.3390/biom10010132

**Published:** 2020-01-13

**Authors:** Neetu Kushwah, Vishal Jain, Dhananjay Yadav

**Affiliations:** 1 Functional Genomics Unit, CSIR-institute of genomics and integrative biology, Mall road, Delhi 110007, India; neetukushwah27@gmail.com; 2 Department of ECE, Carnegie Mellon University, Pittsburgh, PA 15289, USA; 3 Department of Medical Biotechnology, Yeungnam University, Gyeongsan 712-749, Korea

**Keywords:** osmolytes, Alzheimer’s, protein folding/misfolding, Aβ aggregation

## Abstract

Most of the neurological disorders in the brain are caused by the abnormal buildup of misfolded or aggregated proteins. Osmolytes are low molecular weight organic molecules usually built up in tissues at a quite high amount during stress or any pathological condition. These molecules help in providing stability to the aggregated proteins and protect these proteins from misfolding. Alzheimer’s disease (AD) is the uttermost universal neurological disorder that can be described by the deposition of neurofibrillary tangles, aggregated/misfolded protein produced by the amyloid β-protein (Aβ). Osmolytes provide stability to the folded, functional form of a protein and alter the folding balance away from aggregation and/or degradation of the protein. Moreover, they are identified as chemical chaperones. Brain osmolytes enhance the pace of Aβ aggregation, combine with the nearby water molecules more promptly, and avert the aggregation/misfolding of proteins by providing stability to them. Therefore, osmolytes can be employed as therapeutic targets and may assist in potential drug design for many neurodegenerative and other diseases.

## 1. Introduction

Neurodegenerative disorders are described by the accumulation of insoluble aggregates of misfolded proteins in the central nervous system (CNS) such as Parkinson’s disease (PD), which is known by the aggregation of α-synuclein protein [1] and Alzheimer’s disease (AD), which demonstrates intracellular tau and extracellular amyloid-β deposition and hyperphosphorylated tau aggregates, respectively [2,3]. The most frequent form of neurodegenerative disease is AD, which is associated with age and distinguished by premature neurovascular dysfunction, loss of memory, continuous neurodegeneration, and numerous pathogenic mechanisms consisting of neuronal loss and functions and presence of amyloid-β (Aβ) plaques and neurofibrillary tangles in the brain [4,5]. There are two main pathological hallmarks of AD that have been found, which include extracellular amyloid plaques developed by aggregated amyloid-β peptides (Aβ) and intracellular neurofibrillary tangles comprised with polymers of changed tau protein [6]. AD progressively damages the brain structure and its functions like memory and cognition.

Misfolded proteins can be produced by different cellular compartments; together with the cytoplasm nucleus and endoplasmic reticulum (ER), they are competently detached by control systems composed of the ubiquitin (Ub)-proteasome system (UPS), chaperone-mediated autophagy (CMA), and macroautophagy [7]. In multiple pathogenesis of AD, an important role of autophagy has been reported, for example, in generating amyloid plaques from amyloid-β (Aβ) production and accumulation via abnormally regulated amyloid precursor protein (APP) yield and in enhancing the activity of β- and/or γ-secretases, intraneuronal neurofibrillary tangles (NFT), because of tau hyper-phosphorylation and neuronal apoptosis. Dysfunction of the autophagy-lysosome pathway can direct towards Aβ accumulation and the formation of insoluble aggregates and tau oligomers since initiation of autophagy enhances the clearance of both soluble and aggregated appearance of Aβ and tau proteins [8].

Aβ monomers are known as primarily α-helical and random coil in structure. Aβ 42 monomers play an important role in the progression of AD and are extremely prone to aggregation; they produce a wide range of soluble oligomers that differ in size and morphology from dimers to trimers and then up to huge prefibrillar structures. These Aβ oligomers bind with neuronal cells and provoke cell death mediating oxidative stress and phagocytosis. The oligomeric forms of Aβ are known to be the main toxic agents in AD [6]. As protein aggregation and misfolding are the main causes of neurodegeneration in AD, PD, etc., there are some small molecular weight molecules that promote protein folding and avert aggregation in contexts to globular proteins; these molecules are known as osmolytes [9].

Dementia has been estimated to be present worldwide with a high prevalence. There are 24 million cases, and the figure is expected to double every 20 years until at least 2040. As the population worldwide continues to age, the risk per the individual will also increase. Roughly, 5.5 million citizens are affected in the United States, and the global occurrence is expected to be high in comparison with other neurological disorders like PD, which affects approximately seven to ten million people worldwide and is not as common as AD. Therefore, in this review, we mainly focus on the effect of osmolytes on Alzheimer’s because of its rapidly increasing pathogenicity worldwide and because naturally occurring osmolytes have a major effect on toxic forms of Aβ in preventing aggregation and oxidative stress.

## 2. Osmolytes

Osmolytes are organic molecules with lower molecular weight that maintain attributes of the biological fluid. They do so by maintaining the integrity of solution modulating properties like viscosity, melting point, and ionic strength. In aqueous solutions, the strength of the nucleic acids and proteins are significantly affected by these osmolytes [10,11,12]. In other word, osmolytes are naturally occurring organic compounds [13], which upsurge the stability of proteins without disturbing their activity [14]. Generally, unfavorable environmental conditions denature the protein. The accumulation of osmolytes to combat denaturing conditions may protect against the conditions mentioned above. These molecules have ability to protect the functions and stability of the proteins under denaturing/stress conditions and cause refolding of misfolded proteins.

## 3. Classification of Osmolytes

**Figure 1:**
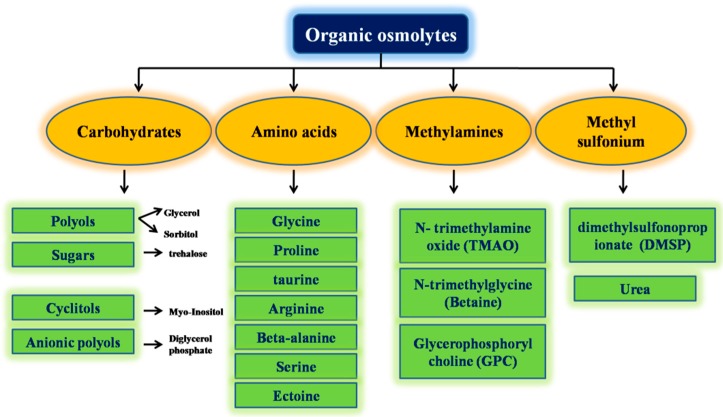
Classification of organic osmolytes.

Osmolytes can be classified as organic osmolytes and were additionally sub-categorized as amino acids, carbohydrates, amines, sulfonium, etc. These protective molecules act as a stabilizing and destabilizing mediator. Urea works as a destabilizing osmolyte, whereas polyols, for example, sorbitol, glucose, sucrose; amino acids and their derived products like betaine, taurine, proline, and glycine; and a few methyl ammonium compounds like sarcosine and trimethylamine N-oxide (TMAO), are categorized as defensive or else stabilizing osmolytes [11,12]. Some frequently used protective osmolytes, like sorbitol, trehalose, betaine, proline, sucrose, TMAO, and so on, can exhibit a destabilizing property on proteins below definite protein-specific conditions (high concentration of osmolyte and/or non-physiological pH range) [10,12]. Below are listed classes of some organic osmolytes (Figure 1).

## 4. Mechanisms of Actions of Osmolytes

**Figure 2:**
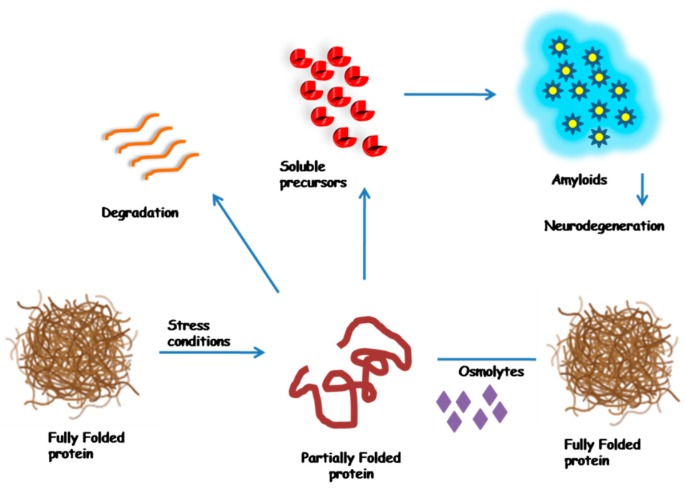
A probable curative mechanism for using osmolytes to prevent misfolded/aggregation. Under stress conditions, the structure of a fully folded protein is compromised due to inadequate folding of protein that may result either in discarded degradation or into the development of soluble precursors to facilitate amyloid formation. Osmolytes can assist in converting partially folded protein back into fully folded protein, thereby restoring proper functions of proteins, leading to the prevention of disease.

Folding of the protein is a progression that is reversible in the environment and osmolytes drive the folding symmetry in the direction of natively folded conformations by increasing the free energy of the unfolded state [15]. Melting temperature (Tm) of many proteins has been shown to increase by the action of osmolytes [16]. Osmolytes perform functions by shifting the properties of solvent in the cellular ambiance, and hence, their occurrence ultimately modifies the strength of these macromolecules [17]. These molecules support the protein in preserving its strength in the aqueous solution and play an important role in retrieving the folded conformation of a denatured protein. Osmolyte moves forward to the folding stability from the unfolded to natively folded conformation through increasing the free energy of the unfolded state. A possible mechanism of osmolytes to prevent misfolded/aggregation under stress conditions has been shown in Figure 2.

Osmolytes provide stability to proteins through osmophobicity, preferential exclusion from protein surfaces, surface tension, and excluded volume. These forces are responsible for the stability of a protein. It is assumed that the assets of the osmolytes force the proteins to fold into a native conformation in spite of the undesirable effect of adverse environmental conditions. Based on the transmission of free energy of amino acid side chain and peptide backbone from water to osmolyte solution, it is thought that the capacity of osmolyte to stabilize protein evolves from the adverse interactions between osmolytes and the functional group, i.e., peptide backbone [18]. To be aware of the high-pressure influences on biochemical systems, essential knowledge about pressure effects on the thermodynamic properties of such osmolytes is significant. The study indicated the high-pressure effects on different biochemical systems where a particular focal point was laid on the effects of pressure on osmolytes such as TMAO, urea, ectoine, glycerol, and glycine as well as the dipeptides acetyl-*N*-methylglycine amide, acetyl-*N*-methylalanine amide, and acetyl-*N*-methyl leucine amide. [19,20]. The study also reported the ability of osmolytes like polyethylene glycol and TMAO for inhibiting of the depolymerization of individual microtubule filaments and that they may potentially play an essential role in in vivo microtubule dynamics [21].

## 5. Osmolyte Prevents Protein Misfolding, Aggregation, and Fibrillization

The viability of cells is retained only when the proteins in them hold their native structure under optimum temperature and pH [14]. In many of the genetic, age-related diseases/pathological conditions, there is a breakdown of misfolded or aggregated proteins. These misfolded proteins are coupled to form a fibrillar arrangement that further leads to amyloid-associated disorders [22]. These pathophysiological circumstances share one title, i.e., the protein conformational diseases. This category has been found to include many neurological disorders including serpin-deficient disorders, AD, Huntington disease, PD, cystic fibrosis, diabetes type 2, transmissible spongiform encephalitis, hemolytic anemia, amyotrophic lateral sclerosis, and dialysis-related amyloidosis [23,24]. Therefore, utilization of naturally occurring organic osmolytes to alter the protein from non-native conformations to its native conformations can be used to prevent various disorders related to misfolding of proteins. Still, the destabilizing osmolytes could be used to eliminate the fibrillar protein structures made inside the cell. Amino acids, lysine, and arginine are frequently used in the solubilization of fibrillar structures and inclusion bodies [25,26]. Thus, osmolytes along with good stabilizers for proteins are also identified as good refolders [10]. Moreover, several proteins are well-known to bind to specific proteins, consequently transforming the native conformation, just like in the process of posttranslational modification [27]. Error in the protein folding pathway or mutation in its gene may lead to misfolded proteins, and these can be recognized as abnormal proteins that are exposed to undergo degradation in the protein quality control (PQC system). Degradation of protein may result in dysfunctional protein [28]. Protein misfolding is another of the most important reasons for protein dysfunction that tends to build up in the endoplasmic reticulum (ER), which is known as a type of deficiency coupled with the trafficking pathway following functional deficiency. Several studies have already reported that when osmolytes were supplemented to the solution, which contains mutant proteins having misfolded conformation, their native function was restored [29,30]. Previous studies have suggested that specific osmolytes can assist the correct folding of misfolded proteins, which in turn may avert their degradation and increase their intracellular function [31,32,33,34,35,36]. The alteration in AQP2 (aquaporin-2) gene causes misfolding of AQP-2 protein that may lead to developing diabetes insipidus in mammals. However, as soon as osmolytes like glycerol (1M) were supplemented in the cell culture medium, glycerol re-established the folded arrangement and consequently the appropriate reshuffle of this protein in the cell [37,38].

In addition to protein misfolding, the other condition where proteins fail to adopt or retain their native state leads to aggregation and fibrillization of proteins. This is one of the major reasons found in the pathophysiology of various neurological and metabolic disorders like PD, AD, Huntington’s disease (HD), type-2 diabetes, prion related encephalopathies, and familial amyotrophic lateral sclerosis (FALS) and in diseases related to repeat expansion and polyglutamine (polyQ) expansion. Studies have already reported that chaperones are effective suppressors of neurological disorders and, consequently, show potential therapeutic targets for disorders related to conformational changes in protein [39,40]. Hence, it is very important to discover some ways or strategies that can lead to avoidance in the development of aggregated/fibrillar structures. The osmolytes can inhibit the protein aggregation/fibrillization by altering the conformational stability and have assisted in the advancement of possible therapeutic strategies aligned with the disorders that arise due to protein misfolding. The study showed that 4-hydroxy-L-proline, L-proline, sarcosine, and TMAO avert fibrillization or aggregation of proteins [41]. Osmolytes like polyol, except erythritol, facilitate the refolding of misfolded or aggregated proteins. These could be used as efficient representative molecules in preventing protein aggregation and in the treatment of numerous aggregation-related devastating diseases [42].

A study has shown that at 3M or above, concentrations of proline initiated the process to avert the accumulation of bovine carbonic anhydrase [43]. An additional study projected the proline as a “protective agent to prevent aggregation of proteins” because it was able to reduce the abnormal interactions between polypeptide chains of protein incredibly early into the pathogenic trail of protein aggregation [44]. The deposition of the polyglutamine-rich variety of huntingtin protein takes place within the nucleus, which is known as a feature of the brain of patients having HD [45]. The study had shown [46] this if 21-day aged mice were given oral administration of 2% of trehalose solution continuously until the day they were killed. There was a reduction in aggregation affinity of the disease related with polyglutamine containing protein huntingtin. This indicated the improvement in loss of motor function and also improved the lifespan of the transgenic HD mouse.

Various literatures have revealed diverse outcomes of osmolytes on the pathway of Aβ aggregation. For instance, trehalose was identified as a probable osmolyte that decreases the Aβ-cytotoxicity by restraining the development of Aβ aggregate [47]. A further study confirmed that sucrose was capable of decelerating the expansion of Aβ fibril. Osmolyte was brought into being to obstruct the racemization reaction of D-aspartic acid [48], which is the major provider to the development of deposits of Aβ [49]. A study attempted to explore the function of osmolytes in the amyloid-coupled aggregation model established on insulin (human) hormone protein. They observed that TMAO, sorbitol, and glycerol resulted in lowering the rate of fibril production by reducing the progression of the unfolding of monomers. The above-mentioned investigational results have indicated an excellent link through volume segregation principle relevant to polymer crowding [38].

## 6. Osmolytes as Therapeutic Target Against Neurological Disorders

During different disease conditions, proteins do not fold into their biochemically active forms leading to the disturbance in biological processes like transport across membranes, protein degradation, and protein folding. Several genetic disorders have been attributed to problems associated with excessive degradation or formation of aggregates in the related proteins. This phenomenon is quite common in neurodegenerative diseases such as Alzheimer’s disease, transmissible spongiform encephalities, serpin deficient disorders, haemolyticanaemia, Huntington disease, cystic fibrosis, diabetes type II, amylotropic lateral sclerosis, Parkinson’s disease, and dialysis related amyloidosis among others. Regulating the brain dimensions is a homeostatic practice wherein the water movement plays an important part in retaining ionic and osmotic balance. It is significant for the appropriate functionality and well-being of the nervous system and is strongly prohibited by a particular cell type called astrocytes, owing to their high and exclusive expression of the water channel, aquaporin-4 [50,51]. Water-influx through AQP4 is initiated by osmolytes that activate an outflow of Cl- and osmolytes by means of some volume-regulated anion channel, and afterwards there is an outflow of water to re-establish the volume [52,53,54,55].

**Figure 3:**
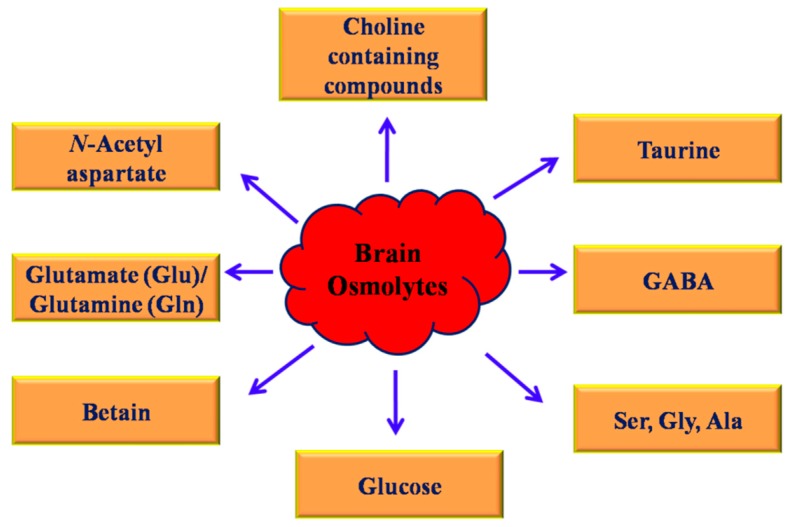
Major classes of the brain osmolytes.

Recently, it has been shown that certain naturally occurring osmolytes can be used to protect these proteins from misfolded conformations leading to prevention of such diseases by virtue of promoting their intracellular functional activity. Reduction and imbalance of osmolytes such as myo-inositol occur due to increasing concentrations of glutamine following astrocyte swelling and the development of low-grade cerebral edema [56]. A common osmolyte such as betaine (N, N, N-trimethylglycine), has played a significant role in the number of clinical reports associating betaine administration with enhanced neuroprotection and cognition [57]. Figure 3 represents various major classes of brain osmolytes. Studies have reported that brain osmolytes like taurine and glycine facilitate the effects of urea on biological activity as urea has a tendency to reduce the k_cat_ and enhance the K_m_ of enzymatic reactions. Betaine (trimethylglycine) regulates plant responses to several stresses together with decreased growth, which is perhaps a part of the plant’s adjustment aligned with stress. γ-aminobutyric acid (GABA) is a main inhibitory neurotransmitter in the brain. Its levels are associated with osmolytes in the visual but not in the sensorimotor region, indicating the regional specificity of modifications in GABAergic tone in hepatic encephalopathy. Glutamate and other osmolytes are expected to be released through receptors and influence synaptic conduction as well as N-methyl-D-aspartate receptor or metabotropic glutamate receptor-dependent synaptic plasticity [13,55,58].

Alzheimer’s disease (AD) is considered a permanent brain disorder that gradually demolishes cognitive functions and ultimately a person’s capacity to execute everyday life tasks and behavior. Memory dysfunctions are one of the initial characteristics of AD, and while it steps forward, deterioration in further cognitive aptitudes such as reduced judgment and alteration in mood start to surface. Eventually people with serious AD cannot speak properly and become entirely reliant on others for their wellbeing. The majority of people having AD have late onset of illness that generally develops after the age of 60. The most important pathological means of AD involve the buildup of amyloid-β (Aβ) peptide in certain areas of the brain [59], and also the occurrence of protein misfolding is very frequent in cells. Accumulation of small peptides amyloid-β (Aβ) in the brains of AD patients is the most established observation regarding the pathological mechanism of AD [60]. Aβ is produced by the proteolysis of an amyloid-β protein precursor (AβPP). AβPP can be cleaved by the three different proteases at three different locations designated as α-, β-, and γ-secretases. Naturally, occurring osmolytes can augment the thermodynamic strength of proteins by providing stability to the natively folded protein conformation, therefore averting aggregation of protein exclusive of perturbing additional cellular processes. Osmolytes might inhibit the development of Aβ oligomers in vivo, consequently prohibiting the evolution of soluble oligomers.

Similarly, Huntington disease (HD) is a progressive neurodegenerative disorder with onset in middle age. Huntington disease is caused by mutation in gene encoding the protein huntingtin1 [61]. Similar to AD, during HD, aggregation of insoluble huntingtin protein aggregates has been observed in different experimental models as well as in brain tissues from patients with Huntington disease [62]. Thus, the identification of a putative therapeutic target that inhibits the formation of polyglutamine aggregates might contribute to the treatment and understanding of polyglutamine diseases like HD.

Small organic molecules or osmolytes have neuroprotective effects in a transgenic mouse model of Huntington disease [63]. Minocycline and creatine retard the progression of pathology and delay mortality [63]. Congo red ameliorates the disease by inhibiting oligomerization of huntingtin [64]. Although the potential therapeutic importance of small molecules that prevent the formation of polyglutamine aggregates is extensively documented, the extreme insolubility of expanded polyglutamines makes it difficult to prepare polyglutamine-containing proteins on a large scale and to search for inhibitors of protein aggregation by in vitro high-throughput screening [65,66].

Correspondingly, several other diseases involve protein aggregation in their pathophysiology, like PD, encephalitis, amyotrophic lateral sclerosis, serpin deficient disorders, hemolytic anemia, cystic fibrosis, and diabetes type 2 [13,67,68,69]. To facilitate the inhibition of protein aggregation, it is important to provide stability to the proteins, which is the main way of preserving their exact form [70]. Therefore, the environment offers a unique way for the entire organism to continue to exist in traumatic situations, facilitating materials like osmolytes. Osmolytes are the molecules that affect osmosis and are soluble in the solution inside a cell or the nearby fluid. They actively participate in preserving cell volume and fluid equilibrium [71]. Several types of osmolytes have been brought to save proteins from denaturation, misfolding, and amyloid development and aggregation in stressed environmental conditions and may play an important part in protecting from life-threatening neurological disorders [72,73,74].

## 7. Conclusions

Stressful environment leads to the generation of the misfolded aggregated structure of the protein that further leads to the generation of neurological disorders. Osmolytes have been found to participate in preventing the aggregation and misfolding of proteins. They can be utilized as curative targets for many neurological disorders, which are primarily associated with the protein misfolding. Fibrillation of protein is liable for a number of amyloidogenic disorders counting diseases like AD, HD, PD, cystic fibrosis, diabetes type 2, and dialysis linked amyloidosis. A detailed understanding of mechanisms of action of osmolytes can lead to the expansion of osmolytes as an efficient curative target molecule and consequently to consistent drug design for the prevention and cure of neurological, genetic, and other diseases caused by protein misfolding/fibrillation/aggregation along with other factors.
